# Comparative Experimental Investigation of Biodegradable Antimicrobial Polymer-Based Composite Produced by 3D Printing Technology Enriched with Metallic Particles

**DOI:** 10.3390/ijms231911235

**Published:** 2022-09-23

**Authors:** Waleed Ahmed, Ali H. Al-Marzouqi, Muhammad Hamza Nazir, Tahir A. Rizvi, Essam Zaneldin, Mushtaq Khan

**Affiliations:** 1 Engineering Requirements Unit, College of Engineering, UAE University, Al Ain P.O. Box 15551, United Arab Emirates; 2 Department of Chemical and Petroleum Engineering, College of Engineering, UAE University, Al Ain P.O. Box 15551, United Arab Emirates; 3 Department of Medical Microbiology & Immunology, College of Medicine, UAE University, Al Ain P.O. Box 15551, United Arab Emirates; 4 Zayed Center for Health Sciences, UAE University, Al Ain P.O. Box 15551, United Arab Emirates; 5 Department of Civil and Environmental Engineering, College of Engineering, UAE University, Al Ain P.O. Box 15551, United Arab Emirates

**Keywords:** antimicrobial, 3D printing, PLA, metallic particles

## Abstract

Due to the prevailing existence of the COVID-19 pandemic, novel and practical strategies to combat pathogens are on the rise worldwide. It is estimated that, globally, around 10% of hospital patients will acquire at least one healthcare-associated infection. One of the novel strategies that has been developed is incorporating metallic particles into polymeric materials that neutralize infectious agents. Considering the broad-spectrum antimicrobial potency of some materials, the incorporation of metallic particles into the intended hybrid composite material could inherently add significant value to the final product. Therefore, this research aimed to investigate an antimicrobial polymeric PLA-based composite material enhanced with different microparticles (copper, aluminum, stainless steel, and bronze) for the antimicrobial properties of the hybrid composite. The prepared composite material samples produced with fused filament fabrication (FFF) 3D printing technology were tested for different time intervals to establish their antimicrobial activities. The results presented here depict that the sample prepared with 90% copper and 10% PLA showed the best antibacterial activity (99.5%) after just 20 min against different types of bacteria as compared to the other samples. The metallic-enriched PLA-based antibacterial sheets were remarkably effective against *Staphylococcus aureus* and *Escherichia coli*; therefore, they can be a good candidate for future biomedical, food packaging, tissue engineering, prosthetic material, textile industry, and other science and technology applications. Thus, antimicrobial sheets made from PLA mixed with metallic particles offer sustainable solutions for a wide range of applications where touching surfaces is a big concern.

## 1. Introduction

Because of environmental concerns, polymeric composite materials have recently gained much attention globally from researchers in different fields such as printing and packaging. Furthermore, the various properties of these materials (thermal, chemical, mechanical, etc.) have been improved by adding fillers [1,2]. The most practiced technique is the addition of minerals as fillers in the composite materials to enhance the antiviral and antimicrobial properties for different applications. Therefore, polymer composite materials are preferred to pure polymers for different applications because of their durability, eco-friendly nature, biodegradability, and cost-effectiveness. Due to the current COVID-19 pandemic, novel and efficient approaches are being practiced globally to resist viruses. In general, COVID-19 has affected nations’ health and economy, and the disease has resulted in 33.3 million infections and more than one million deaths worldwide [3,4]. In the early 20th century, the Spanish flu pandemic spread by the influenza virus infected 500 million people and resulted in approximately 50 million deaths [5,6]. Both influenza and coronaviruses cause respiratory infections, and these types of viruses can be transmitted from a patient to a healthy person through respiratory droplets and contact.

Furthermore, there is some evidence that these viruses (especially SARS-CoV-2) can survive on surfaces for an extended period, depending on the weather conditions [6]. Therefore, touching the surfaces in public places occupied by viruses is one of the leading causes of viral transmission [7]. Thus, contaminated surfaces have been identified as a significant source of the transmission of the virus, especially in hospitals, healthcare centers, and diagnostic centers where frontline workers are in close contact with the patients and inanimate surfaces [8]. Hence, there is a dire need to develop a cost-effective, biodegradable, and environment-friendly antimicrobial polymeric composite that can be applied on different surfaces to reduce the growth and transmission of viruses in the atmosphere and lower the infection rate [9]. Various types of metal nanoparticles and their oxides, e.g., copper (Cu), silver (Ag), cobalt (Co), nickel (Ni), titanium (Ti), and aluminum (Al), have been used in different forms (soluble and insoluble) because of their antiviral and antimicrobial properties [10,11,12]. However, copper and silver have the broadest range of medical devices and product applications. The free ions of these metals can break the membranes of different viruses and eliminate them from the host cell. Furthermore, the nanoparticles of cuprous oxide have shown an excellent tendency against the attachment and entry stages of the hepatitis C virus, proving the virucidal properties of copper nanoparticles [13,14]. 

Copper and silver ionizers are used to control *Legionella* in water supply systems in healthcare settings to reduce nosocomial infections [15]. The efficiency of silver-containing wound dressings has been proven as they reduced cell viability up to 99% [16]. Microporous silica-based composite material was effectively used to remove strontium-90 and cesium-137 from high-level liquid waste produced by reprocessing nuclear spent fuel [17]. The antibacterial activity of silver and copper nanoparticles is much dependent on their stability, size, shape, and capping agent. The agglomeration of silver nanoparticles may occur because of their high reactivity, which can reduce their activity [18]. Antibacterial mechanisms of metal ions and nanoparticles include the following: (i) direct interaction of metal ions released from metal nanoparticles with the cell wall through electrostatic interactions, inhibiting the growth of bacteria; (ii) breakage of lipids, proteins, and DNA cells by reactive oxygen species; (iii) breakage of plasma membrane via the strong binding of metal particles with cells; (iv) direct interference of metal ions with both proteins and DNA, impairing their function and disturbing the cellular metabolism [19]. To resolve this problem, polymeric composite materials may be developed by using other organic and inorganic compounds in the form of fillers, enhancing their stability by changing the structure of their surfaces [20]. The enhancement of the mechanical and thermal characteristics, conductivity, and stability of polymeric materials is well reported in the literature [21]. Different types of cost-effective inorganic materials (e.g., silica, calcium carbonate, kaolin clay, mica, and carbon nanotubes) have been effectively utilized as fillers to enhance the stability of the composites [22,23,24]. Among the fillers mentioned above, silica is the most widely used material to increase the stability of composite materials because of its nonreactivity, higher thermal and chemical stability, abundant availability, and biodegradable nature [25,26]. 

Different techniques have been practiced for developing antimicrobial materials (e.g., spray coating, dip coating, and spin coating) through nano- and ultrafiltration. The latter has been widely employed for wastewater treatment as it removes the suspended particles from wastewater [27]. Currently, 3D printing technology is gaining more interest in developing antimicrobial materials for medical applications as it is the most straightforward, cost-effective, and efficient technique to produce complex-shaped materials with enhanced characteristics that are difficult to attain through conventional fabrication techniques [28,29]. In this additive manufacturing method, 3D materials are developed using the CAD model, and deposition materials are added layer by layer [30,31]. The fused filament fabrication technique is the most used method for developing medical devices [32,33]. This is the most efficient technique as it is suitable for fabricating tiny mechanical parts and provides accurate precision, which is helpful for any changes in the desired product even during the fabrication process. Currently, different techniques, i.e., fused filament fabrication, selective laser sintering (SLS), digital light processing (DLP), and stereolithography (SLA), are being employed for 3D printing globally [34,35,36]. Vidakis et al. [37] investigated the antibacterial efficiency of PLA/AgNp developed through fused filament fabrication against two types of common bacteria for different time intervals and showed that the prepared material caused a significant reduction in bacterial percentage. Yang et al. [38] examined the antibacterial efficiency of wood plastic composites reinforced with copper–zinc alloy particles against *E. coli* and claimed a 90.43% reduction in its growth. Furthermore, both the antibacterial efficiency and the mechanical and thermal properties of 3D printed materials were enhanced, as supported by the literature [39,40].

During the COVID-19 pandemic, the use and implementation of 3D printing technology for medical devices and materials improved significantly. Another study evaluated the performance of a 3D printed prosthesis fitted with a leather glove, nylon, and elastic wire by combining polylactic acid and silicone. The authors claimed that patients’ ability to perform different tasks was improved by using this material [41]. In a study reported by Samuel et al. [32], they developed a novel antibacterial hybrid composite using silicate saponite, phloxine B, and thermoplastics. The material was further utilized for the 3D printing of a lung ventilator, which was used commercially in different countries during the COVID-19 pandemic. Polylactic acid is getting more attention in 3D printing technology because it can be obtained from many renewable resources. Furthermore, it is biodegradable, thermally stable, and reusable [34,35]. Another effective application of 3D printing technology observed during the COVID-19 pandemic was the development of personal protective equipment (e.g., face shields, surgical masks, goggles, and gloves) due to their relative simplicity, low geometrical tolerance requirements, and lower-risk classification within the Food and Drug Administration (FDA) or other regulatory authorities as compared to more complex devices such as ventilators and valves [42,43]. As stated above, copper has excellent antimicrobial characteristics, but its direct use in the medical field is costly. However, it can be used to develop different cost-effective medical devices with enhanced antiviral features. Copper particles are more effective than other materials, e.g., silver, which causes a reaction with the skin in some cases [41]. The effectiveness of 3D printing in the medical field, such as artificial organs for the human body, in terms of cost and antibacterial properties has already been proven by various studies [44,45]. In this study, the antibacterial properties of PLA-based sheets prepared through fused filament fabrication were investigated. Further studies related to mechanical and thermal properties will be performed in the next phase.

In the present study, four types of polymeric samples reinforced with metallic microparticles were processed and produced using 3D printing FFF to explore their antimicrobial characteristics. Polylactic acid (PLA)–copper, PLA–aluminum, PLA–bronze, and PLA–stainless steel with known compositions were prepared, and their efficiencies were tested and compared with a control surface made of steel for different time intervals against common bacteria. The antimicrobial sheet made of PLA and copper presented the best results against all types of tested bacteria just after 20 min. Results showed that the prepared sheet can be an effective antimicrobial agent against Gram-positive and Gram-negative bacteria.

## 2. Results and Discussion

**Figure 1:**
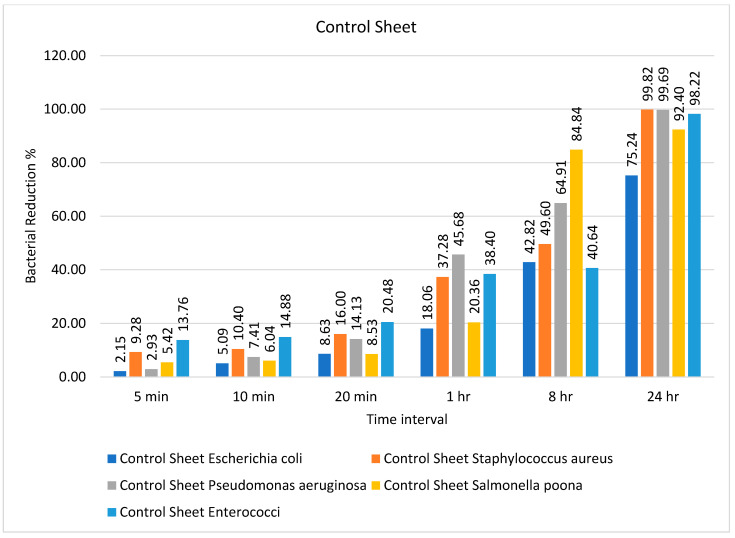
Percentage bacterial reduction for ‘control sheet’ over different time intervals.

Four samples of 3D printed polymeric composite sheets with a known composition of polylactic acid and copper, polylactic acid and aluminum, polylactic acid and bronze, and polylactic acid and stainless steel were prepared and labeled as Samples 1–4, respectively. The antimicrobial activity of the prepared samples was tested against common bacteria that were obtained from certified reference materials, which were preserved in the lab as QC controls. The specific NCTC/ATCC numbers of each organism were mentioned in Section 2. Plastic was taken as a reference surface, as shown in Figure 1, and the efficiency of the antimicrobial sheets was estimated and compared over various time intervals (5 min, 10 min, 20 min, 1 h, 8 h, and 24 h). The initial concentrations of the bacteria are given in Table 1 under the column “inoculum”. The testing protocol was devised according to ISO 22196:2011 [46], and microbiology analytical methods were derived from CCFRA:1:1:4:2003 [47]. The recovered bacterial count on the control sheet over different time intervals is also given in Table 1.

It can be observed from Table 1 that the bacteria showed a decrease in number from 5 min onward, and they continued to show such a reduction up to 24 h except for *Escherichia coli* and *Salmonella* Poona. Both microorganisms continued to be present in considerable numbers even after 24 h, without showing any significant reduction in number. The percentage bacterial reduction on the control sheet is shown in Figure 1. The reduction percentage was significantly lower and showed a random trend ranging from 5 to 20 min for different types of bacteria. However, the rate of reduction started increasing after 20 min, and the maximum decline (99.82%) was observed for *Staphylococcus aureus* after 24 h, as shown in Figure 1.

The number of bacteria on the PLA/copper sample over different time intervals is given in Table 2. The number of recovered bacteria on the PLA/copper sample sheet after just 20 min was significantly lower than that on the control sheet and remained the same up to 24 h. This suggests that the antimicrobial efficiency of the composite sheet produced from PLA/copper against different types of common bacteria was excellent; these results are better than those obtained in a previously published study [48], in which the efficiency of a cellulose-based composite sheet against *E. coli* and *S. aureus* showed a 60% reduction in these bacteria after 24 h against the prepared composite. 

**Figure 2:**
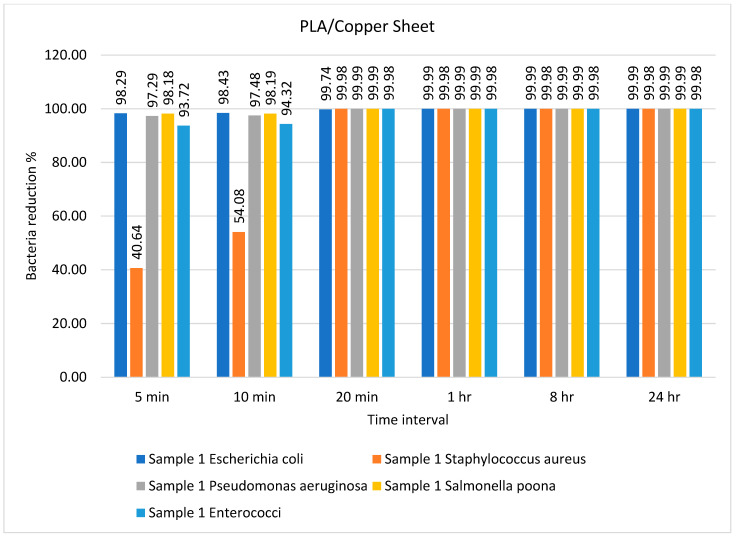
Percentage bacterial reduction for PLA/copper sheet over different time intervals.

The percentage bacterial reduction for the PLA/copper sheet is shown in Figure 2. It can be observed that the maximum decrease in the growth of bacteria was achieved after just 10 min, except for *Staphylococcus aureus*, reaching a reduction of 99.98% after 20 min. These results are comparably better than previous studies described in the literature [49]. Caires et al. [50] studied the antimicrobial efficiency of a graphene oxide- and silver oxide-based composite against different bacteria and reported a 99.4% reduction in growth after 8 h for *Staphylococcus aureus*.

The number of bacteria on the PLA/aluminum-6061 sheet over different time intervals is given in Table 3. Several types of bacteria showed a lower reduction for the PLA/aluminum-6061 sheet as compared to the PLA/copper sheet. This indicates that the composite of PLA and copper had a better tendency to inhibit the growth of bacteria than PLA and aluminum. However, the recovered amount of *S. aureus* and *Enterococci* on the PLA and aluminum sheets was small enough after 1 h, as shown in Table 3. These results agree with those reported by Sajjad et al. [50].

**Figure 3:**
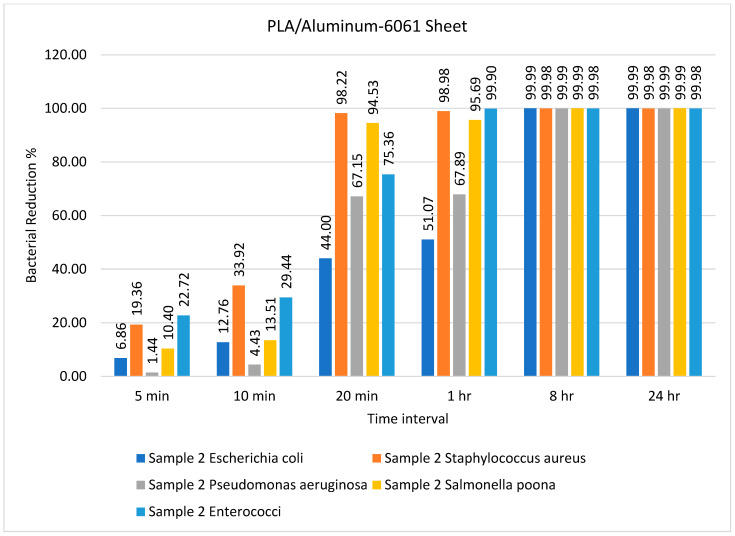
Percentage bacterial reduction for PLA/aluminum-6061 sheet over different time intervals.

The percentage bacterial reduction for PLA/Aluminum-6061 is depicted in Figure 3. The rate of bacterial log reduction was observed as maximum, i.e., 99.99%, after 8 h for all types of bacteria, as shown in Figure 3. In most previous studies, the antimicrobial activity of the prepared composite was tested against one type each of Gram-positive (*Staphylococcus aureus*) and Gram-negative (*Escherichia coli*) bacteria [51]. Goda and colleagues [51] employed *N*-methylene phosphonic acid chitosan/graphene sheets doped with silver nanoparticles as an antimicrobial agent against *Escherichia coli* and *Staphylococcus aureus*, revealing its excellent performance as an antimicrobial agent against these types of bacteria. In the present study, the efficiency of the prepared samples was tested against five different types of bacteria. In another study, Wei and colleagues [52] used urea-derived graphitic carbon nitride sheets doped with silver as a disinfectant for water and *E. coli*.

The number of bacteria on the PLA/bronze sheet during different time intervals is tabulated in Table 4. The PLA- and bronze-based polymeric composite sheets achieved excellent results after only 10 min. Specifically, the recovered amount of all five types of bacteria was exceedingly low and almost completely diminished after 20 min. A similar observation was reported by Alam and colleagues who identified the 3D printed PLA/Ag nanocomposite as the best antimicrobial agent against *E. coli* [53]. Badica and coworkers tested the antimicrobial activity of 3D printed metal-based composites against *E. coli*, *Pseudomonas aeruginosa*, *S. aureus*, *Enterococcus faecium*, *Enterococcus faecalis*, and the yeast strain *Candida parapsilosis* [54]. Another study by Du and colleagues employed a wood-based mesoporous composite doped with silver nanoparticles as an antimicrobial agent to treat wastewater [55]. When the antibacterial efficiency of the prepared composites against *E. coli*, *S. aureus*, and *B. subtilis* was studied, they reported a 99.98% reduction in bacteria after 18 h. This is in sharp contrast to the results presented in this study, which showed that the recovered amount of all five types of bacteria was exceedingly low after 10 min and almost diminished after 20 min (Table 4).

**Figure 4:**
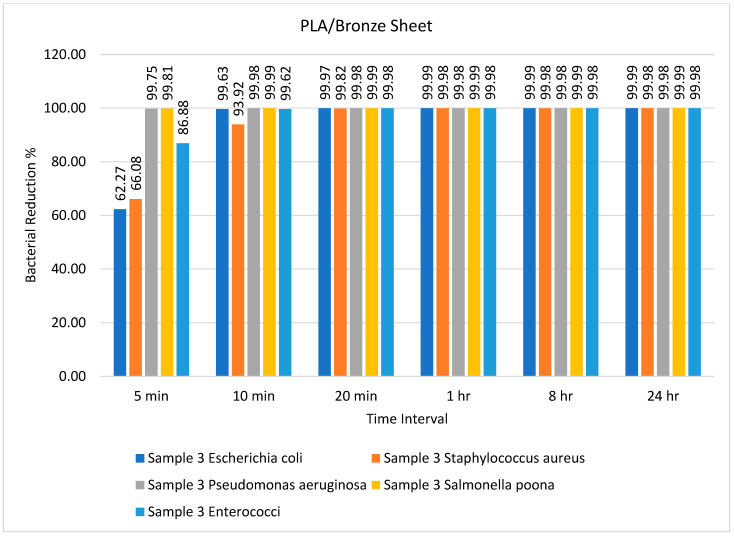
Percentage bacterial reduction for PLA/bronze sheet over different time intervals.

The percentage bacterial reduction for the PLA/bronze sheet is shown in Figure 4. The maximum reductions of 99.75% for *Pseudomonas aeruginosa* and 99.81% for *Salmonella poona* were achieved after just 5 min. Pandey et al. [56] utilized mesoporous Ag/Sn–SnO_2_ composite nanoparticles as an antimicrobial agent against *E. coli* and *Pseudomonas aeruginosa* and successfully inhibited bacterial growth. Comparable results were presented by Nong et al. [57], who used a metal–organic framework-based nanozyme hybrid material as an antimicrobial agent against *Escherichia coli* and *Staphylococcus aureus*.

**Figure 5:**
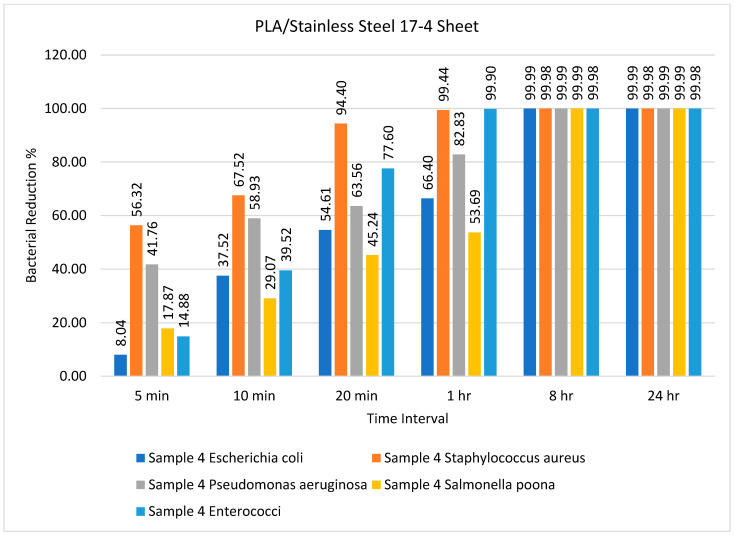
Percentage bacterial reduction for PLA/stainless steel 17-4 sheets over different time intervals.

The number of bacteria on PLA/stainless steel 17-4 sheets over different time intervals is given in Table 5. This sample demonstrated less efficiency as compared to the other samples with respect to time. The bacterial count was minimum after 8 h, which was maintained until 24 h, as shown in Table 5. However, these results contrast with the results of Lou et al. [58]. The percentage bacterial reduction for the PLA/stainless steel 17-4 sheet is illustrated in Figure 5

Figure 5 illustrates that the PLA/stainless steel 17-4 sheet had remarkable efficiency against *Staphylococcus aureus* after 5, 10, and 20 min, achieving almost 100% reduction after 1 h. Furthermore, the bacterial reduction was practically 100% for all bacteria after 8 h and 24 h, as illustrated in Figure 5. In general, we can say that the antimicrobial efficiency of the PLA/copper and PLA/bronze sheets was the same, with some minor differences, whereas the PLA/aluminum-6061 sheet had better efficiency than the PLA/stainless steel 17-4 sheet. The samples were tested against different bacteria for different time intervals, i.e., 5 min, 10 min, 20 min, 1 h, 8 h, and 24 h, to analyze the efficiency of the sheets. After 8 h, the maximum reduction in bacterial amount, i.e., 99.8%, was observed for all prepared sheets except for the reference sheet, and this did not change for the remainder of the test. The order of efficiency can be expressed as PLA/copper ≥ PLA/bronze > PLA/aluminum-6061 > PLA/stainless steel 17-4. 

### 2.1. Bacterial Elimination and Evaluation 

**Figure 6:**
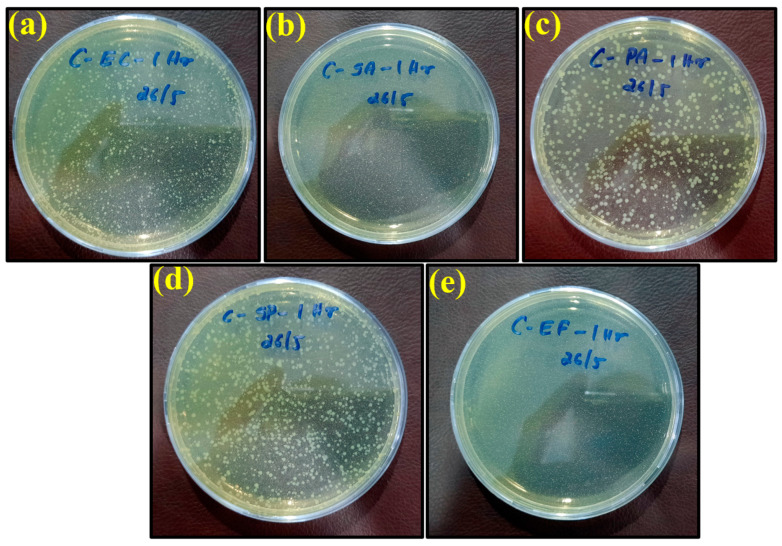
Images of remaining bacteria on the control surface after cultivation for 1 h: (**a**) *E. coli*; (**b**) *S. aureus*; (**c**) *P. aeruginosa*; (**d**) *S.* Poona; (**e**) *Enterococci*.

**Figure 7:**
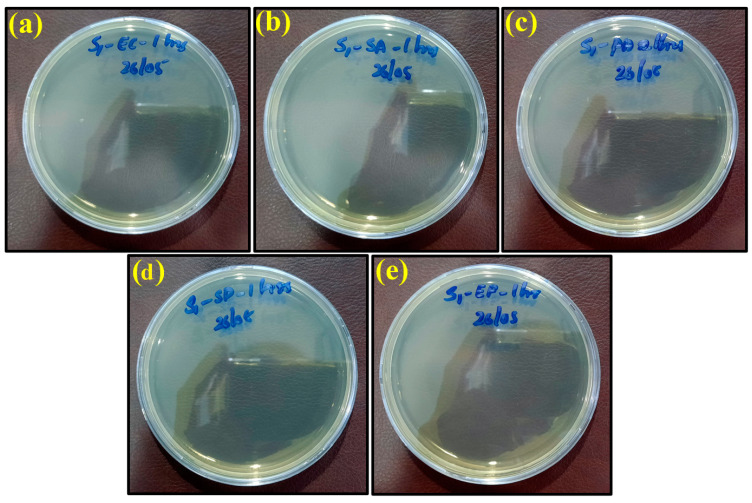
Images of remaining bacteria on PLA/copper sheet after cultivation for 1 h: (**a**) *E. coli*; (**b**) *S. aureus*; (**c**) *P. aeruginosa*; (**d**) *S.* Poona; (**e**) *Enterococci*.

**Figure 8:**
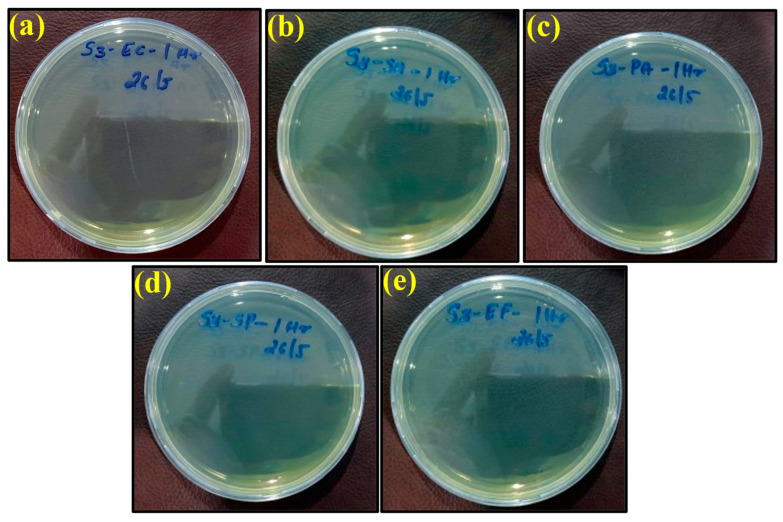
Images of remaining bacteria on PLA/bronze sheet after cultivation for 1 h: (**a**) *E. coli*; (**b**) *S. aureus*; (**c**) *P. aeruginosa*; (**d**) *S.* Poona; (**e**) *Enterococci*.

The plate count procedure was used to determine the antimicrobial efficiencies of the ‘control sheet’, PLA/copper sheet, and PLA/bronze sheet as shown in Figure 6a–e, Figure 7a–e, and Figure 8a–e, respectively, according to their results described in the previous section. 

The specimens were kept in petri dishes for longer exposure times (8 and 24 h). They left some dried liquid marks on the surface, with no color change from yellow observed. In fact, only *Pseudomonas aeruginosa* had a slight greenish shade. The yellow tint may be because of the environmental light at the time of image capture. Growth was observed for all bacteria, i.e., *E. coli*, *S. aureus*, *P. aeruginosa*, *S.* Poona, and *Enterococci*, on the surface of the control petri dishes over different time intervals, as shown in Figure 6. These results indicate that the plastic control sheet had no antimicrobial efficiency against these bacteria.

However, the appearance of bacteria started to diminish after exposure to the PLA/copper sheet and PLA/bronze sheet, as shown in Figure 7 and Figure 8, respectively, validating their excellent antimicrobial efficiency. This is believed to be due to the ability of copper and bronze nanoparticles to coagulate proteins and inhibit bacterial growth [59]. Moreover, bacterial membrane proteins may have bound to the copper and bronze nanoparticles, thus interfering with the synthesis of peptidoglycan and hindering cell-wall synthesis. Such a scenario for efficiently preventing the growth of *E. coli*, *S. aureus*, and other bacteria has previously been reported [60]. 

The maximum antimicrobial efficiency of the PLA/copper sheet after 1 h was 99.99%, 99.98%, 99.99%, 99.99%, and 99.98% against *Escherichia coli*, *Staphylococcus aureus*, *Pseudomonas aeruginosa*, *Salmonella* Poona, and *Enterococci*, respectively. The antibacterial effectivity of the PLA/bronze sheet was similar after 1 h. Furthermore, when the prepared antimicrobial sheets were separated from the inactivated bacteria, the bacteria regained their activities.

### 2.2. Comparison with Other Materials

A comparison of the present study with previously developed antimicrobial composites from different materials is provided in Table 6. In most studies, the antibacterial efficiency was tested against common Gram-positive and Gram-negative bacteria, i.e., *S. aureus* and *E. coli*, as shown in Table 6. The present results are compared in pictorial form with the previous data in Hamid et al. [61] tested the antibacterial activity of a PMMA denture base material modified with ZrO_2_ nanoparticles against *Candida albicans* by varying the concentrations of antimicrobial agents over different time intervals. They claimed the best results with 5% of material after 30 days. In another study reported by Ansari et al. [62], the antimicrobial activity of material prepared from leaf extracts was tested. They demonstrated that the maximum efficiency was achieved after 24 h against common bacteria. Ghanem et al. [63] tested the antibacterial activity of polycaprolactone films containing modified graphene against *S. aureus* and claimed a 76% reduction in density of these bacteria after 24 h. Wang et al. [64] prepared lignin-based composites modified with silver nanoparticles and utilized them as an antibacterial agent against *E. coli* and *S. aureus*. They achieved a 99.9% reduction in these bacteria after 5 min. Benigno et al. [65] tested the antibacterial properties of low-density polyethylene and multiwalled carbon nanotube-based composites against *E. coli* via diffusion. No bacterial colonies were found after 1 h.

## 3. Materials and Methods

Additive manufacturing (AM) encompasses a variety of fabrication technologies. The most utilized method is material extrusion (ME), in which a material filament is fed into the extrusion system and heated near the polymer’s melting temperature. The end effector feeds and fuses the new material layer to the previous one [76]. By fabricating metal parts via ME, the ease of operation, safety, and waste reduction are greatly improved. By utilizing ME technology, the disadvantages mentioned above can be mitigated to produce relatively inexpensive metal parts within a unique research area that is narrowly studied [77]. This method involves fusing a polymer matrix and metal powder to make a filament denoted as a metal–polymer composite (MPC) [78]. The material utilized for the current study was an MPC in a 3D printing filament form. Filaments are metal powders encased in a binder of environmentally friendly, biodegradable, and carbon-neutral polymers (PLA) [79]. Such an operation is safe and does not result in any powdered metal exposure. The antimicrobial sheets were prepared using 3D printing because it allows a flexible design and can print more complex designs faster than traditional manufacturing processes. Furthermore, it produces minimal waste, and it is a more cost-effective and advanced technology to produce lightweight parts. Experimental results showed that the 3D printing parameters, such as printing temperature, bed temperature, extrusion speed, and printing speed, were very close to the PLA 3D printing setting, except for the use of a hard steel printing nozzle due to the high hardness of the metallic particles, which led to wear of the normal nozzle and deformation at the nozzle head, thus disturbing the 3D printing consistency. The filaments used in the study were produced by Virtual Foundry LLC (Stoughton, WI, USA). Four types of filaments were used in this study with a base binder (PLA) through fused filament fabrication: copper/PLA [80], aluminum 6061/PLA [81], bronze/PLA [82,83], and stainless steel 17-4 /PLA [81]. Polylactic acid polymer was used in this study, and the four types of antimicrobial sheets were prepared using this polymer through fused filament fabrication. PLA was selected for developing the antimicrobial sheets because it is easy to use, cost-effective, environment-friendly, and biodegradable in nature with less warping issues, making PLA filaments the perfect material for 3D printing. The primary source of composite filaments is metallic 3D filaments developed to extrude metal particles with a thermoplastic binder into continuous filaments suitable for use in any fused 3D printer. Metal-filled filaments are produced by mixing polymers such as PLA with metal microparticles such as stainless steel, aluminum, copper, and bronze at different mixing ratios. Therefore, the filament is enriched with a high concentration of metallic particles, which provides the composite filament with a greater density than standard plastics. In fact, the metallic filaments utilized in this investigation have been applied for different industrial applications to make metallic parts through a sintering process. Thus, the filaments are enriched with a high concentration of metallic particles. In general, such composite filaments enriched with metallic particles are printed using open-source 3D printers, allowing the option to change the printing setting whenever required, especially when different types of printers from different suppliers are used.

The composition of these composites is described below.

- Sample 1: Copper/PLA

The copper/PLA filament contained 90% copper metal by weight and had a density of 4.7 g/cc.

- Sample 2: Aluminum 6061/PLA

The aluminum 6061/PLA filament contained 65% Al 6061 metal by weight and had a density of 1.54 g/cc.

- Sample 3: Bronze/PLA

The CuSn/PLA filament contained 90% Bronze metal by weight and had a density of 4.5 g/cc.

- Sample 4: Stainless steel 17-4 /PLA

The SS 17-4/PLA filament contained around 85% SS 17-4 metal by weight and had a density of 3.0 g/cc. 

Pure stainless steel does not possess any antibacterial properties; however, the antibacterial properties of stainless steel can be enhanced by various techniques as stated in the literature. Wang et al. [84] enhanced the antibacterial behavior of stainless steel 304 by depositing silver through an electrodeposition method. Di cerbo et al. [85] enhanced the antibacterial activity of stainless steel 288 using nanotechnological surface coatings. Shuai et al. [86] investigated the increase in the antibacterial properties of stainless steel 17-4 using heat treatments. Furthermore, the antibacterial activity of stainless steel can be increased by modifying its surface chemistry and adding metal nanoparticles to its surface [87].

Each composite consists of micro metal powder and the polymer matrix produced through a mixing process by an extruder [88,89]. The spooler pulls the extruded material from the extrusion nozzle at a constant linear travel rate, while optionally allowing spooling of the material, where the spool speed is usually greater than the tension roller’s speed.

The sample (40 mm × 40 mm × 1 mm) was designed using CAD software Fusion 360 and sliced using Ultimaker Cura 4.10 (Utrecht, the Netherland), an open-source slicing application for 3D printers [90]. 

**Figure 9:**
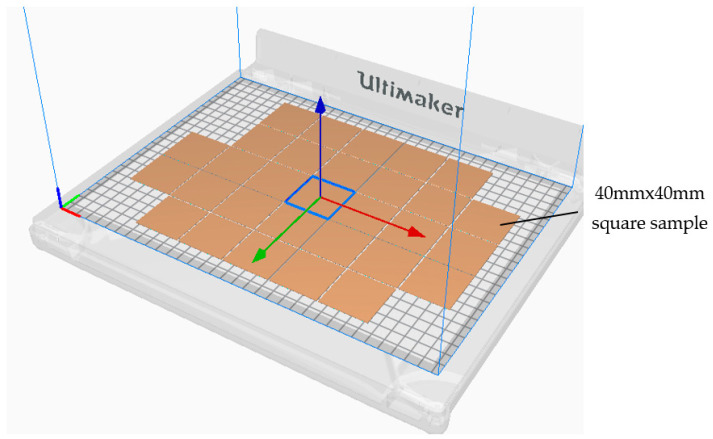
The slicing process of the square samples.

The time needed to produce one sample was 8 min, consuming a filament length of 2.85 mm, requiring 8 g of material filament for the copper/PLA material, for example. Although the antimicrobial tests required 25 samples to cover the microbe types and the incubation time, 30 samples were produced to keep spare samples and were tested once. Furthermore, any unreliable results were excluded and repeated. The 30 3D printed samples took around 4 h to prepare the CAD design and the slicing software, along with 13 h for production time, consuminf 226 g of the CU/PLA filament (approximately 7.88 m). The Cura slicing of the square sample is illustrated in Figure 9.

**Figure 10:**
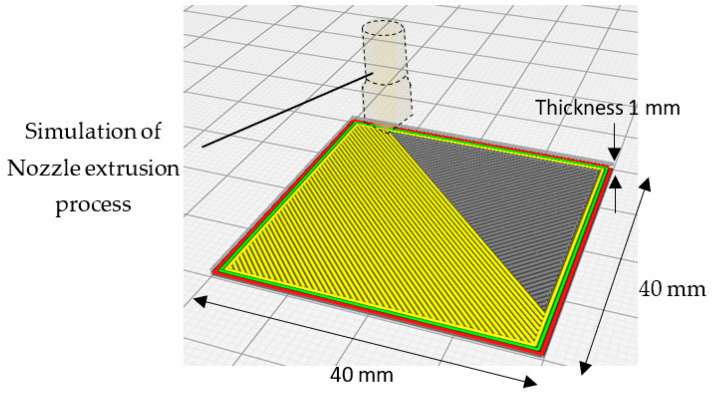
**Slicing** Simulation of the 3D printing process with the slicing features determined by the slicer Cura.

The simulation of the 3D printing process with the slicing features determined by slicer Cura is illustrated in Figure 10. In general, the 3D printed samples were chosen to fit the testing containers, whereas the thickness was considered to maintain proper stiffness of the samples to avoid any unnecessary deformation that could affect their condition during and after the printing process.

**Figure 11:**
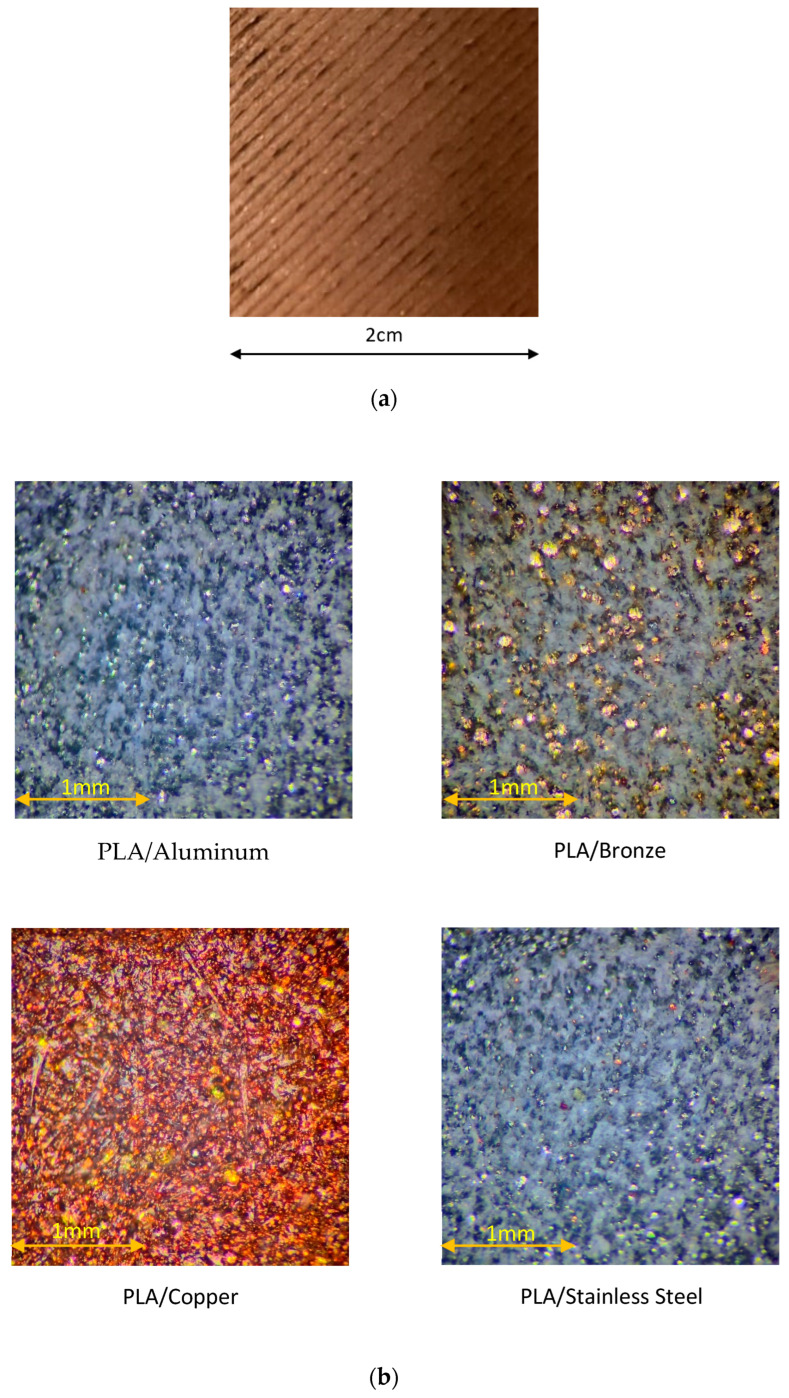
(**a**) Surface of 3D printed CU/PLA composite sample. (**b**) Microscopic images of 3D printed PLA composites with different metal particles.

A macroscopic surface image of the 3D printed CU/PLA composite sample is shown in Figure 11a.

Microscopic images of 3D printed PLA composites with different metal particles are shown in Figure 11b.

**Figure 12:**
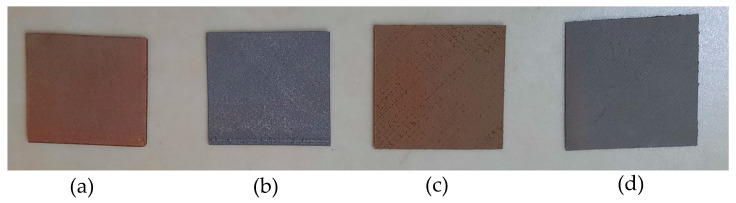
Four types of 3D printed samples: (**a**) Cu/PLA; (**b**) SS 17-4/PLA; (**c**) CuSn/PLA; (**d**) Al 6061/PLA.

The configuration of the slicer Cura setting was as follows: layer height, 0.2 mm; infill pattern lines; infill density, 100%; no support; no adhesion type; speed, 45 mm/s; 100% fan cooling. Due to the printing bed’s smooth surface, the sample’s back side was softer than its front side. Therefore, the smooth side of the 3D printed sample was always used for conducting antimicrobial testing. The four types of prepared 3D printed samples are shown in Figure 12.

The 3D printer Ultimaker UM S5 was used to print the testing samples. It was specially designed to print using composite materials on the 3D printer Ultimaker S5 at a maximum temperature of 300 °C. The 3D printer was developed for the composite materials of third-party material suppliers that can wear out the standard Ultimaker UM S5 Core Head AA. Therefore, they should be printed using the print core CC, and we applied a hardened steel nozzle sized at 0.6 mm using Ultimaker print core CC 0.6. The printing nozzle temperature was maintained at 210 °C while the printing bed temperature was kept at 50 °C; the bed was covered using a layer of blue painter’s tape or glue sticks to achieve maximum adhesion, and the printing flow rate was set at 135%.

The filament was preheated at 60 °C using a warming chamber placed before the feeding gear to minimize any filament bending as it came off the spool. As the filament passed through the warmer, the memory of the filament was reset for ease of printing.

### Antimicrobial Testing and Standards

Different methods and standards have been practiced to test the antimicrobial activity of materials, e.g., agar-based and diffusion methods, which can be further categorized into disc diffusion [91], well diffusion [92], disc volatilization [93], agar spot diffusion, and the parallel streak method [94,95]. The agar disc diffusion testing method is one of the oldest methods (developed in 1940) used for general routine testing. The Clinical and Laboratory Standards Institute has published several approved methods for testing bacteria and yeast [26,96]. These methods are considered standards for antimicrobial susceptibility testing (AST). They are used to calculate the minimum inhibitory concentration (MIC) on agar plates (agar dilution) or in broth microdilution or macro dilution medium for different types of bacteria [97]. Although this method cannot accurately test all the fastidious microbes, several modifications have been made to test different pathogens, e.g., streptococci, *Haemophilus influenzae*, *Haemophilus parainfluenzae*, *Neisseria gonorrhoeae*, and *Neisseria meningitidis*, using specific culture media, different incubation conditions, and interpretive criteria for inhibition zones [98]. 

The antimicrobial gradient procedure uses the principles of both dilution and diffusion techniques. The Etest (bioMérieux, Marcy–L’Étoile), MIC Test Strip (Liofilchem Inc., Waltham, MA, USA), M.I.C. Evaluator (Oxoid, Basingstoke, UK), and Ezy MIC Strip (HiMedia Laboratories Pvt. Ltd., Mumbai, India) are commercially available versions of these techniques. The method is based on the possibility of developing a concentration gradient of the antimicrobial material tested in an agar medium. The significant advantage of using this method is its simplicity and cost-effectiveness. Automated and semiautomated devices such as the VITEK and VITEK 2 systems, MicroScan WalkAway System, Trek Diagnostic Systems, and BD Phoenix System are also used in clinical laboratories to identify the bacteria in a fast and reliable way. The VITEK and VITEK 2 systems are automated devices used for the AST of Gram-negative and Gram-positive bacteria. The VITEK 2 System is the advanced version of VITEK. VITEK can process 32 to 120 test cards, while VITEK 2 can simultaneously process 60 to 120 cards [99]. 

The MicroScan Walkaway System is another commercially available automatic equipment that detects the enzymatic activity of bacteria using fluorogenic substrates and pH indicators. Furthermore, it can process 96 panels at once [100]. The Trek Diagnostic System is a fluorometer-based automated system that can process unlimited panels, and it can be used for both Gram-positive and Gram-negative bacteria [101]. The BD Phoenix System was first introduced in 2003, reducing the duration needed for preparing test panels. It has many incubators that can process 99 panels simultaneously. The results of MIC are generally available for 6–16 h. It is also suitable for both Gram-positive and Gram-negative microorganisms [102]. Furthermore, different types of general bacteria are typically derived from the American Type Culture Collection (ATCC) and Nonprofit Culture Collection (NCTC) repositories to check the antimicrobial activities of different materials against these bacteria. Some general bacteria that are commonly tested against other antimicrobial materials and surfaces derived from the organizations mentioned above are *Escherichia coli* (ATCC 10536) [103], *Pseudomonas aeruginosa* (NCTC 10662) [104], *Staphylococcus aureus* (NCTC 6571), *Salmonella* Poona (NCTC 3940) [105,106], and *Enterococcus faecalis* [107]. 

The antimicrobial activity of surfaces can be analyzed by following the three major standards suggested by Japan (JIS Z2801:2010), Europe (ISO 22196:2011), and recently the United States (US EPA). According to the Japanese standard, the definition of antimicrobial activity is an inhibition of the growth of bacteria on the surface of the material. However, ISO 20743 considers both inhibition and death of bacteria on the surfaces [108]. These methods are adapted to assess the antimicrobial activity and efficiency for plastic, nonporous, and hard surfaces. The standard proposed by the United States Environmental Protection Agency (EPA) has gained more attention than the abovementioned methods because it provides the equations and conditions to test antimicrobial activity and normalized procedures to study the effect of biocidal cleaning liquids on nonporous surfaces. A material is said to be a sanitizer if 99.9% of bacteria are killed within 1 h. On the other hand, the Japanese and European standards do not set an antibacterial activity threshold but rather give a framework for standardized antimicrobial activity quantification; the range of items they cover is more significant, making benchmarking more challenging [49,109].

Another standard, i.e., ISO 22196, has been developed to test the activity of bacteria and viruses on plastic surfaces for a time interval of 24 h. Further modifications have been made to this method to make it applicable to other nonporous surfaces. It is an excellent approach to establishing the antimicrobial activity of a surface. This has become one of the industry standards among several tests for the antibacterial activity of surfaces [46]. The quantitative and precise assessment of antimicrobial surfaces (e.g., plastics, metals, and ceramics) is conducted according to the JIS Z 2801 standard. This method has various real-world applications in different fields, ranging from healthcare centers to household consumer companies. This is the most adopted method in the United States, and it has become an industry standard. However, this method complicates the identification of an ideal control surface [110]. The BS ISO 22196:2011 standard measures the antimicrobial activity on plastic surfaces and paint films that are not light-activated. This standard commonly uses *Escherichia coli* or *Staphylococcus aureus* [111]. The bacteria were measured using the pour plate technique according to CCFRA 1.1.4:2003. The entire test method was developed in-house.

## 4. Conclusions

The antimicrobial properties of polylactide acid (PLA) enhanced with four metals, i.e., copper (Cu), bronze, stainless steel (SS), and aluminum (Al), were investigated by mixing 90% Cu, 65%Al, 85% SS, and 90% bronze with a known amount of PLA via the metal extrusion method. Antimicrobial sheets were synthesized using printing technology, an effective and fast technique for introducing metal ions into the cluster and preparing the desired material with enhanced properties. All prepared sheets were tested against Gram-positive and Gram-negative bacteria according to the standards by varying the time intervals. All antibacterial sheets led to an excellent reduction in bacteria compared to a plastic reference surface, i.e., 99.98% for PLA/bronze after 20 min, 99.9% for PLA/Al-6061 after 8 h, and 99.97% for PLA/SS17-4 after 20 min. However, the polymeric 3D printed sheet made from Cu and PLA showed the maximum bacterial reduction of 99.99% after just 20 min against all types of bacteria tested. The results of these antibacterial sheets are effectively higher than those published earlier for different antimicrobial materials due to the percentage of the metallic particles used. Furthermore, the use of 3D printing technology for the preparation of antimicrobial polymeric composites could enhance their characteristics and performance through the consistent extrusion process. Therefore, this technology may be helpful for further investigations and applications in the biomedical, food, space, and textile fields, as well as other science and technology applications like recycling of waste materials [112,113,114] and implementing nanotechnology for better performance [115]

## Figures and Tables

**Table 1:** Bacterial count on control sheet during different time intervals.

Sample	Type of Bacteria	Inoculum	Bacterial Amount					
			5 min	10 min	20 min	1 h	8 h	24 h
Control sheet	*Escherichia coli*	9500	9296	9016	8680	7784	5432	2352
	*Staphylococcus aureus*	5000	4536	4480	4200	3136	2520	9
	*Pseudomonas aeruginosa*	7500	7280	6944	6440	4074	2632	23
	*Salmonella* Poona	9000	8512	8456	8232	7168	1364	684
	*Enterococci*	5000	4312	4256	3976	3080	2968	89

**Table 2:** Bacterial count on PLA/copper sheet over different time intervals.

Sample	Type of Bacteria	Inoculum	Bacterial Amount					
			5 min	10 min	20 min	1 h	8 h	24 h
Sample 1 PLA/copper	*Escherichia coli*	9500	162	149	25	1	1	1
	*Staphylococcus aureus*	5000	2968	2296	1	1	1	1
	*Pseudomonas aeruginosa*	7500	203	189	1	1	1	1
	*Salmonella* Poona	9000	164	163	1	1	1	1
	*Enterococci*	5000	314	284	1	1	1	1

**Table 3:** Bacterial count on PLA/aluminum-6061 sheet over different time intervals.

Sample	Type of Bacteria	Inoculum	Bacterial Amount					
			5 min	10 min	20 min	1 h	8 h	24 h
Sample 2 PLA/Al	*Escherichia coli*	9500	8848	8288	5320	4648	1	1
	*Staphylococcus aureus*	5000	4032	3304	89	51	1	1
	*Pseudomonas aeruginosa*	7500	7392	7168	2464	2408	1	1
	*Salmonella* Poona	9000	8064	7784	492	388	1	1
	*Enterococci*	5000	3864	3528	1232	5	1	1

**Table 4:** Bacterial count on PLA/bronze sheet over different time intervals.

Sample	Type of Bacteria	Inoculum	Bacterial Amount					
			5 min	10 min	20 min	1 h	8 h	24 h
Sample 3 PLA/bronze	*Escherichia coli*	9500	3584	35	3	1	1	1
	*Staphylococcus aureus*	5000	1696	304	9	1	1	1
	*Pseudomonas aeruginosa*	7500	15	1	1	1	1	1
	*Salmonella* Poona	9000	17	1	1	1	1	1
	*Enterococci*	5000	656	19	1	1	1	1

**Table 5:** Bacterial count on PLA/stainless steel 17-4 sheet over different time intervals.

Sample	Type of Bacteria	Inoculum	Bacterial Amount					
			5 min	10 min	20 min	1 h	8 h	24 h
Sample 4 PLA/stainless steel	*Escherichia coli*	9500	8736	5936	4312	3192	1	1
	*Staphylococcus aureus*	5000	2184	1624	280	28	1	1
	*Pseudomonas aeruginosa*	7500	4368	3080	2733	1288	1	1
	*Salmonella* Poona	9000	7392	6384	4928	4168	1	1
	*Enterococci*	5000	4256	3024	1120	5	1	1

**Table 6:** Comparison of the present study with the different antimicrobial composites.

Composites	Type of Bacteria	Killing Rate	Ref.
PLA/GO 5%	*S. aureus*, *E. coli*	100% (24 h) for *S. aureus* and *E. coli*	[66]
PPY/CuO	*S. aureus*, *E. coli*	100% (8 h)	[67]
Ag/PPY	*S. aureus*, *E. coli*	92.6% (24 h)	[68]
SiO_2_/PANI	*P. aeruginosa*	100% (12 h)	[69]
MWCNT/PANI	*S. aureus*, *E. coli*	99.9% (24 h)	[70]
Cu_2_O/rGO	*E. coli*	70% and 65% for 18 h	[71]
Stainless Steel coated with ZrO_2_/ZnO/TiO_2_	*S. aureus*, *E. coli*	81.2% and 72.4% after 12 h	[72]
Stainless steel doped with TiO_2_	*E. coli*	99.9% after 4 h under UV	[73]
Stainless steel modified with peptide	*S. aureus*, *E. coli*	56.9% after 3 h	[74]
Stainless steel coated with derived antimicrobial peptide	*V. natriegens* and C. farmer (marine bacteria)	99.79% and 99.33% after 24 h	[75]
PLA/copper	*E. coli*, S. aureus	99.99% and 99.98% after 1 h	Present study
PLA/aluminum	*E. coli*, S. aureus	99.99% (8 h) and 98.98% (1 h)	Present study
PLA/bronze	*E. coli*, S. aureus	99.99% and 99.98% (1 h)	Present study
PLA/stainless steel	*E. coli*, S. aureus	99.99% and 99.98% (8 h)	Present study

Ag = silver, MWCNT = multiwalled carbon nanotube, PANI = polyaniline, CNPs = copper nanoparticles, PLA = polylactic acid, HNT = halloysite nanotube, GO = graphene oxide, PPY = polypyrene, PVK = polyvinyl carbazole, ZnO = zinc oxide, TiO_2_ = titanium dioxide, ZrO_2_ = zirconium dioxide. According to a comparison of the data in the literature with the present study, we can conclude that the antimicrobial composites developed herein using 3D printing technology showed excellent antimicrobial activity over a minimum time frame.

## Data Availability

Not applicable.
